# Capsule Size Alters the Timing of Metabolic Alkalosis Following Sodium Bicarbonate Supplementation

**DOI:** 10.3389/fnut.2021.634465

**Published:** 2021-02-19

**Authors:** India Middlebrook, Joe Peacock, Daniel J. Tinnion, Nicholas K. Leach, Nathan P. Hilton, Bryan Saunders, S. Andy Sparks, Lars R. Mc Naughton

**Affiliations:** ^1^Sports Nutrition and Performance Research Group, Department of Sport and Physical Activity, Edge Hill University, Ormskirk, United Kingdom; ^2^Applied Physiology and Nutrition Research Group, University of São Paulo, São Paulo, Brazil; ^3^Department of Sport and Movement Studies, Faculty of Health Science, University of Johannesburg, Johannesburg, South Africa

**Keywords:** buffering, gastrointestinal disturbance, performance, acid base balance, palatability

## Abstract

**Introduction:** Sodium bicarbonate (NaHCO_3_) is a well-established nutritional ergogenic aid that is typically ingested as a beverage or consumed in gelatine capsules. While capsules may delay the release of NaHCO_3_ and reduce gastrointestinal (GI) side effects compared with a beverage, it is currently unclear whether the capsule size may influence acid–base responses and GI symptoms following supplementation.

**Aim:** This study aims to determine the effects of NaHCO_3_ supplementation, administered in capsules of different sizes, on acid–base responses, GI symptoms, and palatability.

**Methods:** Ten healthy male subjects (mean ± SD: age 20 ± 2 years; height 1.80 ± 0.09 m; weight 78.0 ± 11.9 kg) underwent three testing sessions whereby 0.3 g NaHCO_3_/kg of body mass was consumed in either small (size 3), medium (size 0), or large (size 000) capsules. Capillary blood samples were procured pre-ingestion and every 10 min post-ingestion for 180 min. Blood samples were analyzed using a radiometer (Radiometer ABL800, Denmark) to determine blood bicarbonate concentration ([${\text{HCO}}_{3}^{-}$]) and potential hydrogen (pH). GI symptoms were measured using a questionnaire at the same timepoints, whereas palatability was recorded pre-consumption.

**Results:** Capsule size had a significant effect on lag time (the time [${\text{HCO}}_{3}^{-}$] changed, *T*_lag_) and the timing of peak blood [${\text{HCO}}_{3}^{-}$] (*T*_max_). Bicarbonate *T*_lag_ was significantly higher in the large-sized (28 ± 4 min) compared with the small-sized (13 ± 2 min) capsules (*P* = 0.009). Similarly, *T*_max_ was significantly lower in the small capsule (94 ± 24 min) compared with both the medium-sized (141 ± 27 min; *P* < 0.001) and the large-sized (121 ± 29 min; *P* < 0.001) capsules. The GI symptom scores were similar for small-sized (3 ± 3 AU), medium-sized (5 ± 3 AU), and large-sized (3 ± 3 AU) capsules, with no significant difference between symptom scores (*F* = 1.3, *P* = 0.310). Similarly, capsule size had no effect on palatability (*F* = 0.8, *P* = 0.409), with similar scores between different capsule sizes.

**Conclusion:** Small capsule sizes led to quicker *T*_lag_ and *T*_max_ of blood [${\text{HCO}}_{3}^{-}$] concentration compared to medium and large capsules, suggesting that individuals could supplement NaHCO_3_ in smaller capsules if they aim to increase extracellular buffering capacity more quickly.

## Introduction

Sodium bicarbonate (NaHCO_3_) is an extensively researched nutritional ergogenic aid shown to be particularly effective in improving short-duration (~1–10 min), high-intensity exercise performance (1–3). Supplementation with NaHCO_3_ serves to enhance endogenous bicarbonate buffering capacity by inducing temporary elevations in extracellular bicarbonate concentrations and, resultantly, enhancing the efflux of hydrogen cations (H^+^) from the skeletal muscle. Consequently, an improved H^+^ efflux attenuates muscular fatigue and has been shown to positively impact multiple performance measures such as total work done (4), power output (5), and time to exhaustion (6) and recovery between exercise bouts (7).

The ergogenic potential of NaHCO_3_ is widely acknowledged (8), but some individuals suffer adverse gastrointestinal symptoms (9, 10) (GIS) that may be deleterious to performance (11, 12). Recently, some authors have attempted to find strategies to alleviate the severity of GIS by using delayed release (2019) and enterically coated capsules (13). This strategy builds on the concept of reducing GIS by delaying the release of HCO_3_ into the stomach, thereby limiting carbon dioxide production that occurs when NaHCO_3_ is ingested (14, 15). At present, these coatings make this ergogenic strategy expensive. Indeed the most frequently used ingestion strategy is gelatine capsule delivery of NaHCO_3_. This is both a cheap alternative, improves the palatability compared to the traditional solution, and widely used by athletes and researchers.

Encapsulation may result in reductions in the HCO_3_ lost in the stomach and bring about comparable acid–base changes using smaller doses than required from aqueous delivery (15). There is, however, a suggestion that encapsulation may impair or slow down bicarbonate availability through decreased gut transit time (16) changing the optimal pre-exercise ingestion time. Additionally, while the gastro-resistant properties of different capsule forms and their subsequent effects on bicarbonate bioavailability have begun to be elucidated (10, 13, 17), the effects of the physical properties of capsules, such as their overall size (and therefore surface area), on bicarbonate bioavailability remain unclear.

In the pharmaceutical industry, the bioavailability of a substance is carefully considered as part of delivery vehicle testing and is affected by size, surface area, and surface area/volume of the capsule. Furthermore, there is a direct relationship between the surface area of a substance and its dissolution rate; specifically, an increase in total surface area of a delivery vehicle in contact with the gastrointestinal fluids causes an increase in the dissolution rate (18). Indeed the dissolution of substances from capsules is a complex function of four key factors: (1) the rate of dissolution of the capsule shell, (2) the rate of penetration of gastrointestinal fluids into the gastrointestinal mass, (3) the rate at which the mass disaggregates in the gastrointestinal fluids, and (4) the rate of dissolution of the dispersed substance particles (18). Such factors are rarely considered in the delivery of ergogenic aids despite these processes being highly variable and subject to potentially large inter-individual variation (19). Given the considerable evidence of the ergogenic effects of NaHCO_3_ and the widespread use of capsules as an ingestion strategy, understanding how capsule size (and therefore surface area) impacts bioavailability is of high importance for optimizing pre-exercise ingestion timing (20). Therefore, the aim of this study was to determine the effects of NaHCO_3_ supplementation administered in different-sized capsules on blood acid–base responses, GIS, and palatability.

## Method

### Participants

Ten recreationally active male participants, with the following (mean ± SD) characteristics, volunteered for this study: age, 20 ± 2 years; height, 1.8 ± 0.2 m; body mass, 78.0 ± 11.9 kg. All participants undertook regular (≥3 days·wk^−1^) exercise for at least 30 min per session. Following medical screening, all participants were deemed healthy, free from GI disorders, and not taking any nutritional supplements or prescription medication. The protocol was explained in full, and questions were answered before the participants gave written informed consent to participate in the study. The study was approved by the Departmental Research Ethics Committee.

### Study Design

The participants visited the laboratory on three separate occasions after an overnight fast and at the same time of day. The visits were separated by between 24 and 72 h to allow acid–base balance variables to return to normal (21, 22). The participants maintained their habitual diet before experimental testing (23) and refrained from alcohol ingestion and strenuous exercise at least 24 h before each visit. During the initial visit, height (Seca, Germany) and body mass (Holtain, UK) were recorded before the participants consumed 300 mg NaHCO_3_/kg body mass in gelatine capsules (Bulk Powders™, Colchester, UK). This dose was chosen based on previous findings of improved exercise performance and is a dose widely recognized to be ergogenic within the literature (3, 4, 24, 25). Capsule sizes were administered using a repeated-measures crossover design, following the use of a Latin square to determine trial order allocation for participants (26). The three trials used either standard small (size 3), medium (size 0), and large (size 000) capsules. Each capsule contained 0.4, 0.8, and 1.6 g NaHCO_3_, and the mean number of capsules consumed was 59 ± 9, 29 ± 4, and 15 ± 2, which equated to a total capsule surface area for the bolus of 23.3 ± 3.5, 20.7 ± 3.1, and 16.4 ± 2.5 cm^2^ for the small, medium, and large capsule size, respectively. The capsules were consumed with 400 ml of water which was at room temperature (18°C). Capsule palatability was recorded immediately post-ingestion. The participants remained seated for 180 min while blood acid–base responses and GI symptoms were monitored throughout.

### Acid–Base Responses

During the experimental protocol, exposure response was established through mapping the time course of blood [${\text{HCO}}_{3}^{-}$] and potential hydrogen (pH). Fingertip capillary blood procurement was chosen as it is a method widely used in exogenous buffering intervention literature (2, 4, 17, 20, 22) and is a recognized method for blood gas analysis. Capillary blood was drawn pre-ingestion and then post-ingestion every 10 min for 3 h, an established protocol for examining acid–base changes following exogenous buffer ingestion (3, 10, 13). Samples were collected in 100-μl heparin-coated glass capillary tubes (Radiometer Medical Ltd., Denmark) using an aseptic technique and were analyzed immediately using a blood gas analyzer (Radiometer ABL800 BASIC, Denmark). These data were then used to determine the peak in [${\text{HCO}}_{3}^{-}$] change (*C*_max_), the absolute change in [${\text{HCO}}_{3}^{-}$] (Δ*C*_max_), the time to reach *C*_max_ (*T*_max_), the area under the concentration–time curve (AUC), and the time lag (*T*_lag_). The *T*_lag_ was defined as an increase in [${\text{HCO}}_{3}^{-}$] beyond normal daily variability (13).

### Gastrointestinal Symptoms and Palatability

At the same time points, the GI symptoms were measured using a nine-item questionnaire which included stomach cramping, flatulence, nausea, belching, stomach ache, diarrhea, vomiting, bowel urgency, and stomach bloating (27). Each symptom was measured on an 11-point scale, whereby “0 = no symptom” and “10 = severe symptom.” Palatability was recorded immediately post-ingestion using a nine-point hedonic scale, where “1 = extremely dislike” and “9 = extremely like” (28).

### Statistical Analysis

All data were assessed for normality by the Shapiro–Wilk test and by visual inspection of the normality plots (29). Blood acid–base responses (${\text{HCO}}_{3}^{-}$ and pH) and GI symptoms were analyzed using two-way (condition × time) analysis of variance (ANOVA) with repeated measures. A general linear model ANOVA was used to analyze absolute acid–base values [peak blood [${\text{HCO}}_{3}^{-}$], time-to-peak blood [${\text{HCO}}_{3}^{-}$], peak blood pH, time-to-peak blood pH, and area under the curve (AUC)], GI symptoms, and perceived palatability. Two-tailed statistical significance was set at *p* < 0.05. Effect sizes were reported as partial eta-squared (ηp^2^) and are described as trivial (<0.20), small (ηp^2^ = 0.20–0.49), moderate (ηp^2^ = 0.50–0.79), and large (≥0.80), respectively (30).

## Results

### Blood Bicarbonate Responses

**Figure 1:**
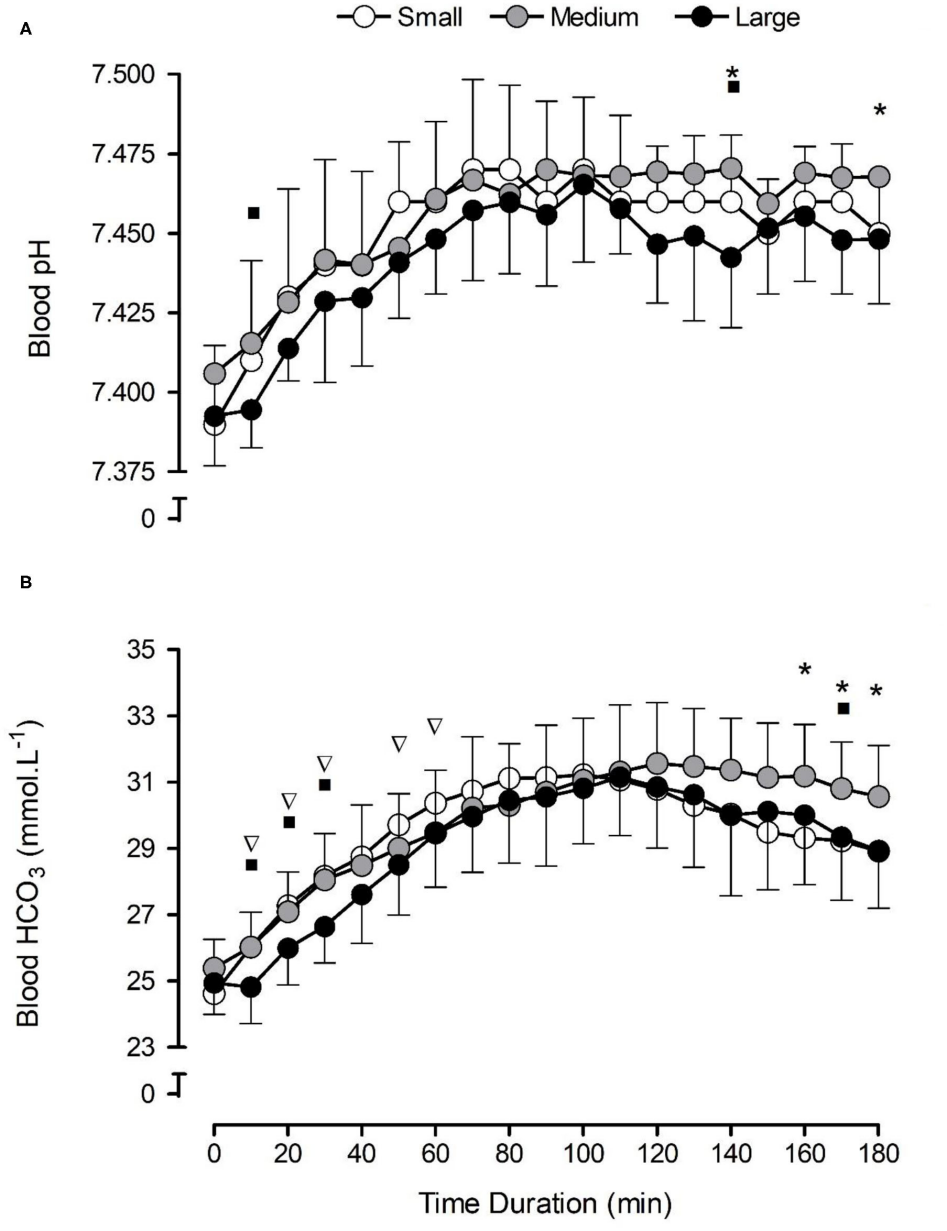
Mean (±SD) temporal blood pH **(A)** and bicarbonate concentration [${\text{HCO}}_{3}^{-}$] **(B)** responses following the consumption of 0.3 g·kg^−1^ body mass NaHCO_3_ in small-, medium-, and large-sized capsules. *a condition × time interaction between small and medium capsules, where *p* < 0.05. ∇a condition × time interaction between small and large capsules, where *p* < 0.05. ■a condition × time interaction between medium and large capsules, where *p* < 0.05.

There were significant increases in blood [${\text{HCO}}_{3}^{-}$] (*F* = 93.2, *p* < 0.001, ηp^2^ = 0.91) in all NaHCO_3_ conditions compared with pre-consumption values (Figure 1B). The capsule size had no significant effect on [${\text{HCO}}_{3}^{-}$] (*F* = 2.3, *p* = 0.151, ηp^2^ = 0.21) post-consumption, although a significant condition × time interaction was observed (*F* = 3.3, *p* = 0.014, ηp^2^ = 0.27), suggesting that the large capsules changed [${\text{HCO}}_{3}^{-}$] more slowly in the initial part of the post-ingestion period, and the medium-sized capsule sustained [${\text{HCO}}_{3}^{-}$] for a longer time (Figure 1B).

**Figure 2:**
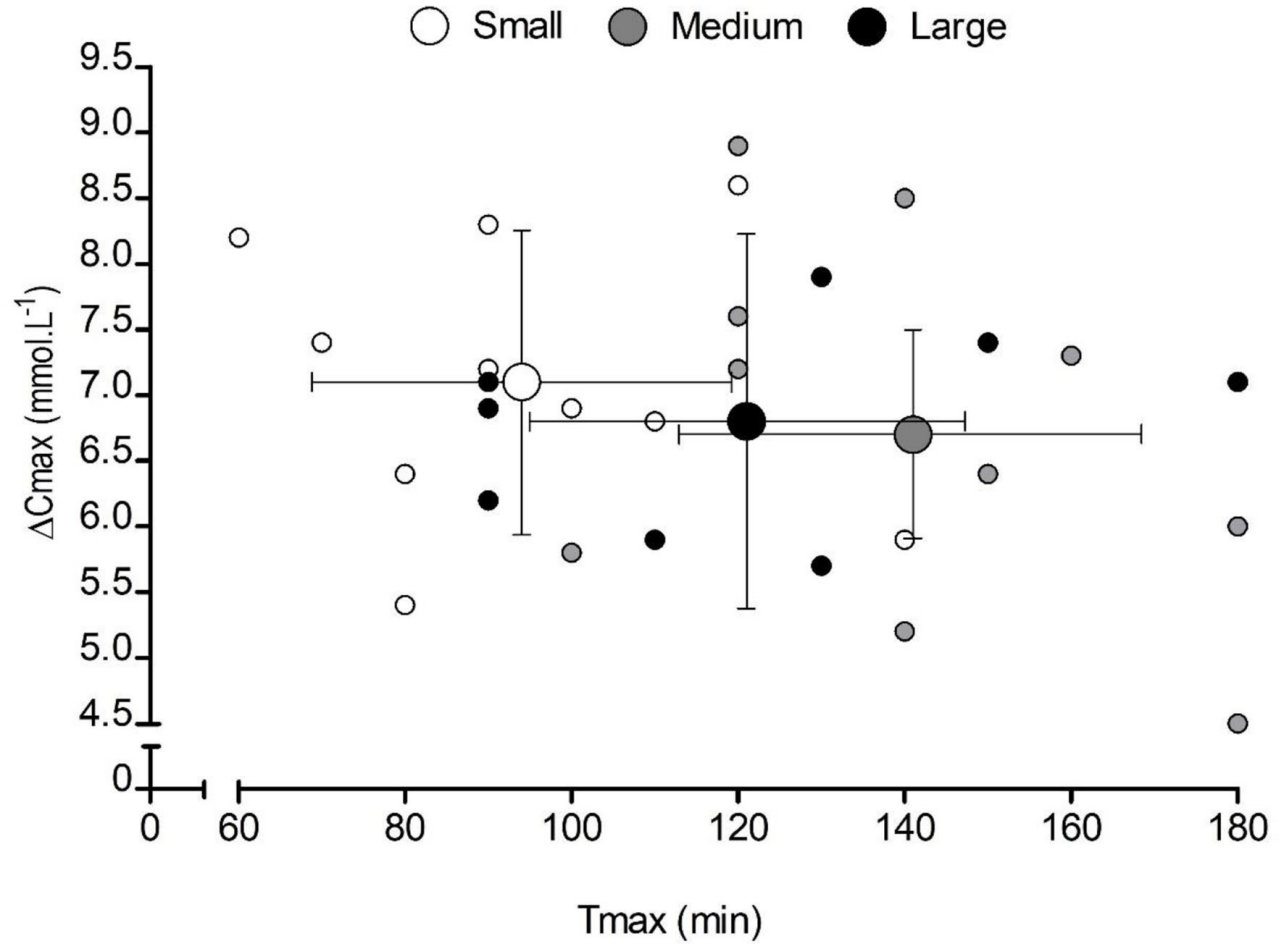
Mean (±SD) and individual peak changes in blood bicarbonate concentration [${\text{HCO}}_{3}^{-}$] (Δ*C*_max_) following the consumption of 0.3 g·kg^−1^ body mass NaHCO_3_ in small-, medium-, and large-sized capsules. Small markers represent individual responses, and large markers represent the mean data for each capsule condition. The X and Y whiskers represent the SD of the sample in each condition for time-to-peak [${\text{HCO}}_{3}^{-}$] (*T*_max_ and Δ*C*_max_, respectively).

Capsule size also had a significant effect on *T*_lag_ (*F* = 3.8, *p* = 0.043, ηp^2^ = 0.30), with significantly longer times in the large-sized (28 ± 4 min) compared with the small-sized (13 ± 2 min) capsules (*p* = 0.009). Similarly, capsule size had a significant effect on *T*_max_ (*F* = 157.6, *p* = 0.000, ηp^2^ = 0.94), with significantly shorter times in the small capsule compared with both the medium-sized (*p* = 0.000) and the large-sized (*p* = 0.000) capsules (Table 1). No significant differences were observed for *C*_max_ (*F* = 0.6, *p* = 0.574, ηp^2^ = 0.06), Δ*C*_max_ (*F* = 0.3 *p* = 0.731, ηp^2^ = 0.03), or AUC (*F* = 2.1, *p* = 0.148, ηp^2^ = 0.19) between conditions (Table 1). There appeared to be a large inter-individual variability in response to capsule ingestion (Figure 2).

**Table 1:** Mean (SD) bicarbonate kinetic variables following the consumption of 0.3 g·kg^−1^ body mass NaHCO_3_ in small-, medium-, and large-sized capsules.

**Variable**	**Small**	**Medium**	**Large**
*T*_lag_ (min)	13 ± 2	22 ± 6	28 ± 4^a^
*C*_max_ (mmol·L^−1^)	31.7 ± 1.7	32.1 ± 1.5	31.8 ± 1.4
Δ*C*_max_ (mmol·L^−1^)	7.1 ± 1.1	6.7 ± 1.4	6.8 ± 0.8
*T*_max_ (min)	94 ± 24^b^	141 ± 27^b^	121 ± 29^b^
AUC (mmol·min·L^−1^)	5,316 ± 256	5,373 ± 264	5,239 ± 263

*T_lag_, lag time; C_max_, peak bicarbonate concentration; ΔC_max_, change in peak bicarbonate concentration; T_max_, time-to-peak bicarbonate concentration; AUC, area under the curve*.

a *Significant difference between the large and small capsules (p < 0.05)*.

b *Significant difference between all capsules (p < 0.001)*.

### Blood pH Responses

Blood pH increased in all NaHCO_3_ conditions (*F* = 41.5, *p* < 0.001, ηp^2^ = 0.82) compared with pre-consumption values (Figure 1A). Capsule size had a significant effect on blood pH (*F* = 3.9, *p* = 0.040, ηp^2^ = 0.30) overall, although no significant condition × time interaction was shown for blood pH (*F* = 0.9, *p* = 0.628, ηp^2^ = 0.09; Figure 1A). There were no significant differences in either peak blood pH (*F* = 1.5, *p* = 0.249, ηp^2^ = 0.14) and time-to-peak blood pH (*F* = 1.9, *p* = 0.181, ηp^2^ = 0.17) between conditions.

### Gastrointestinal Symptoms and Palatability

**Figure 3:**
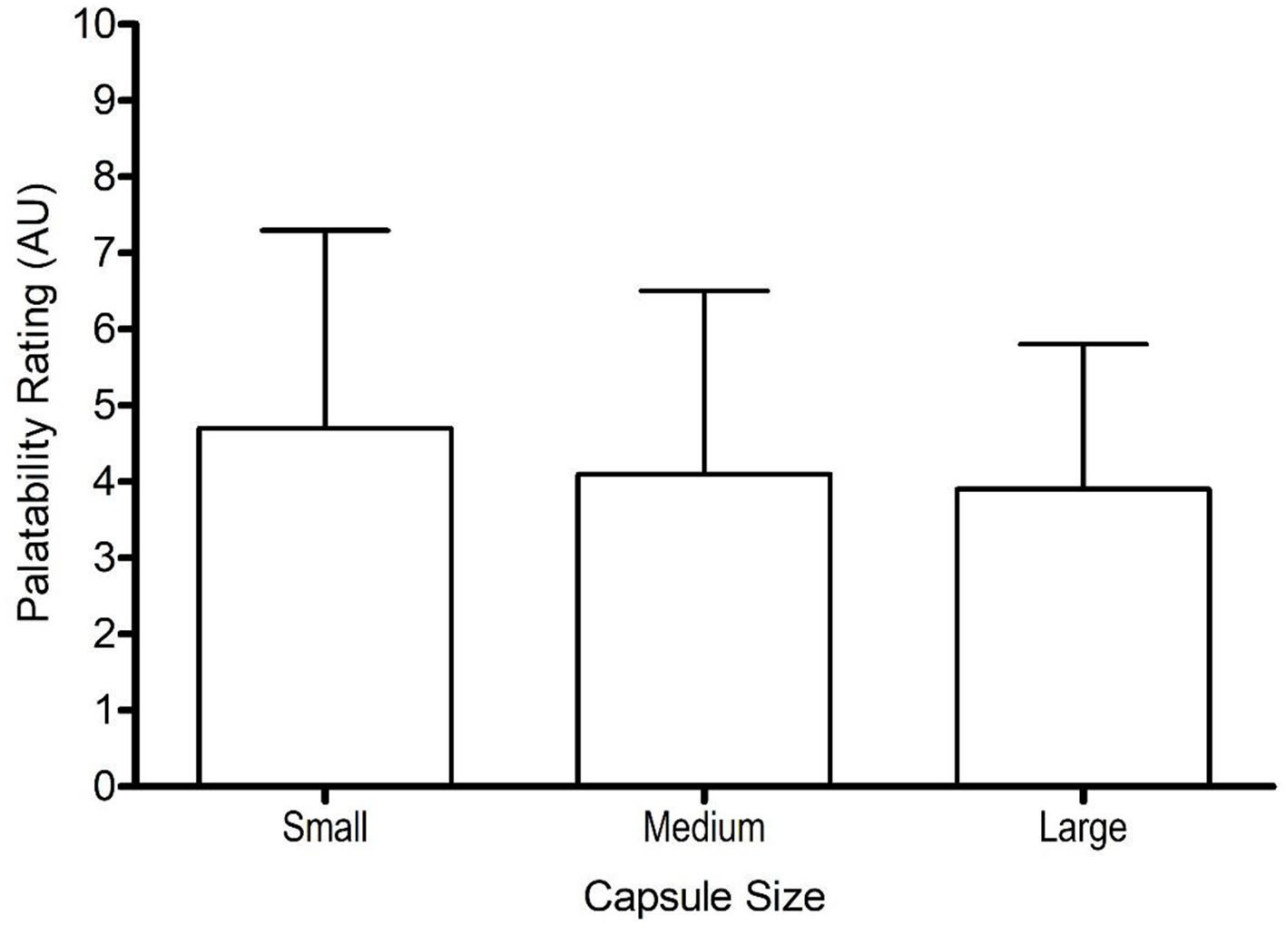
Mean (±SD) palatability scores following the consumption of 0.3 g·kg^−1^ body mass NaHCO_3_ in small-, medium-, and large-sized capsules.

Gastrointestinal symptom scores were similar for small-sized (3 ± 3 AU), medium-sized (5 ± 3 AU), and large-sized (3 ± 3 AU) capsules, with no significant difference between symptom scores (*F* = 1.3, *p* = 0.310, ηp^2^ = 0.12). Similarly, capsule size had no effect on palatability (*F* = 0.8, *p* = 0.461, ηp^2^ = 0.08), with similar scores between different capsule sizes (Figure 3). The palatability scores ranged from 1–9, 1–9, and 1–7 for small, medium, and large capsule, respectively.

## Discussion

This study showed that different capsule sizes led to differences in *T*_lag_ and *T*_max_ of blood [${\text{HCO}}_{3}^{-}$] without affecting the absolute increases in circulating ${\text{HCO}}_{3}^{-}$ or AUC of the increases over 180 min. Since *T*_lag_ (vs. large capsules) and *T*_max_ was shorter (vs. medium and large capsules) for small capsules, and palatability was similar, albeit also without affecting GI symptoms, this suggests that smaller capsules may be a better form of ingestion for individuals wishing to increase their extracellular buffering capacity more quickly. Those using capsules to administer NaHCO_3_ should also be cognizant of the trade-off in palatability and participant comfort due to the inverse relationship between capsule size and the number of capsules needed to deliver a potentially ergogenic dose (31). Despite the mean differences in ${\text{HCO}}_{3}^{-}$ kinetics when smaller capsules are consumed, we observed considerable individual variability in responses, similar to those previously reported (19, 32–34).

Alternative forms of NaHCO_3_ ingestion will lead to different pharmacokinetic profiles, with the most common forms in solution or gelatine capsules, with apparently different ${\text{HCO}}_{3}^{-}$ kinetics (33). Enterically coated and delayed release forms also lead to different ${\text{HCO}}_{3}^{-}$ kinetics compared to gelatine capsules (10, 13, 17). These novel data now show that different sizes of gelatine capsules lead to different blood ${\text{HCO}}_{3}^{-}$ kinetics, with quicker increases and time to reach peak values with smaller capsules. Previously, the dissolution rates for individual size 0 and 3 gelatine capsules have been observed to be similar at around 100 s (35). However, in the present study, the large differences between the number of capsules ingested between capsule size conditions results in considerable differences in the total surface area of the ingested substance. Consequently, the greater total surface area of the smaller capsules is likely to liberate their contents quicker. There were no differences between the medium- and large-sized capsules shown here. For those intending to ensure that the start of exercise coincides with *C*_max_, these data suggest that individuals could adapt the capsule size in which they ingest NaHCO_3_ depending on when they can supplement. The present study also standardized the temperature of the fluid ingested with the capsules, but consuming hotter fluids is likely to reduce *T*_max_, and colder fluids are likely to increase *T*_max_ (35). At present, no studies have considered the temperature of the fluid on the pharmokinetics of extracellular buffers such a NaHCO_3_, but athletes and sports nutrition practitioners should be aware that this is likely to alter the expected time duration at which NaHCO_3_ should be ingested prior to exercise. It would be of interest to determine whether enterically coated versions of these capsules also lead to different and more favorable ${\text{HCO}}_{3}^{-}$ kinetics following ingestion.

Side effects associated with NaHCO_3_ ingestion include nausea, vomiting, GI discomfort, diarrhea, and headache (4). There have been some suggestions that minimizing neutralization of stomach acids due to the increased NaHCO_3_ load might lead to reduced GI discomfort and increased circulating ${\text{HCO}}_{3}^{-}$ (15). This explains why enterically coated and delayed release forms of NaHCO_3_ reduce the incidence and severity of GI disturbances compared to gelatine capsules (10, 13, 17). Despite the different ${\text{HCO}}_{3}^{-}$ profiles presented here, there were no differences in the side effect symptom scores between the different capsule sizes, suggesting that individuals need not concern themselves with side effects when choosing which size of the gelatine capsule to use for NaHCO_3_ supplementation. Nonetheless, further work should elucidate whether enterically coated versions of these different capsule sizes can reduce their side effects since discomfort associated with NaHCO_3_ can be ergolytic to exercise performance (12).

A limitation of this study is that we only analyzed the time course of blood ${\text{HCO}}_{3}^{-}$ and pH kinetics following NaHCO_3_ supplementation in different capsule sizes. It could have been interesting to determine whether different exercise performance responses were shown between the capsules. Nonetheless, it could be hypothesized that similar performance improvements would be shown seen if exercise was performed at TTP since there were no differences between capsule sizes for peak ${\text{HCO}}_{3}^{-}$ change, absolute ${\text{HCO}}_{3}^{-}$ at peak, and ${\text{HCO}}_{3}^{-}$ AUC. It is possible that performance differences might be found should standardized ingestion times be employed prior to exercise since *T*_max_ was different between capsule sizes. Therefore, it is important to ensure that individual responses to the specific type of capsules that are being used are determined in order to optimize the pre-exercise timing of their ingestion.

In conclusion, small capsule sizes led to quicker *T*_lag_ and *T*_max_ of blood [${\text{HCO}}_{3}^{-}$] compared to medium and large capsules, without affecting absolute increases in circulating ${\text{HCO}}_{3}^{-}$ or AUC. The palatability and GI symptoms were similar between all capsule sizes. Individuals could supplement NaHCO_3_ in smaller capsules if they aim to increase extracellular buffering capacity more quickly.

## Data Availability Statement

The raw data supporting the conclusions of this article will be made available by the authors, without undue reservation.

## Ethics Statement

The studies involving human participants were reviewed and approved by Department of Sport and Physical Activity Research Ethics Committee, Edge Hill University. The patients/participants provided their written informed consent to participate in this study.

## Author Contributions

IM, SS, and LM designed the study. IM, DT, NL, and NH collected the data. IM, NH, and SS analyzed the data. All the authors contributed to the interpretation of data, writing of the manuscript, have read and approved the final version of the manuscript, and agreed with the final order of presentation of the authors.

## Conflict of Interest

The authors declare that the research was conducted in the absence of any commercial or financial relationships that could be construed as a potential conflict of interest.
